# Navigating the challenges of imposter participants in online qualitative research: lessons learned from a paediatric health services study

**DOI:** 10.1186/s12913-024-11166-x

**Published:** 2024-06-12

**Authors:** Pakhi Sharma, Steven M McPhail, Sanjeewa Kularatna, Sameera Senanayake, Bridget Abell

**Affiliations:** 1 Australian Centre for Health Services Innovation and Centre for Healthcare Transformation, School of Public Health and Social Work, Queensland University of Technology, 60 Musk Avenue, Kelvin Grove, Brisbane, QLD 4059 Australia; 2 Digital Health and Informatics Directorate, Metro South Health, Brisbane, QLD Australia; 3 Health Services and Systems Research, Duke-NUS Medical School, Singapore, Singapore

**Keywords:** Imposter participants, Fraud, Online research, Paediatric neurodevelopmental care, Research integrity

## Abstract

**Background:**

The growth in online qualitative research and data collection provides several advantages for health service researchers and participants, including convenience and extended geographic reach. However, these online processes can also present unexpected challenges, including instances of participant fraud or scam behaviour. This study describes an incident of participant fraud identified during online focus group discussions and interviews for a PhD health services research project on paediatric neurodevelopmental care.

**Methods:**

We aimed to recruit carers of Australian children with neurodevelopmental disorders. Potential participants were recruited via a publicly available social media advert on Facebook offering $50 AUD compensation. Those who expressed interest via email (*n* = 254) were sent a pre-interview Qualtrics survey to complete. We identified imposters at an early stage via inconsistencies in their self-reported geographical location and that captured by the survey as well as recognition of suspicious actions before, during and after focus group discussions and interviews.

**Results:**

Interest in participation was unexpectedly high. We determined that all potential participants were likely imposters, posing as multiple individuals and using different IP addresses across Nigeria, Australia, and the United States. In doing so, we were able to characterise several “red flags” for identifying imposter participants, particularly those posing as multiple individuals. These comprise a combination of factors including large volumes and strange timings of email responses, unlikely demographic characteristics, short or vague interviews, a preference for nonvisual participation, fixation on monetary compensation, and inconsistencies in reported geographical location. Additionally, we propose several strategies to combat this issue such as providing proof of location or eligibility during recruitment and data collection, examining email and consent form patterns, and comparing demographic data with regional statistics.

**Conclusions:**

The emergent risk of imposter participants is an important consideration for those seeking to conduct health services research using qualitative approaches in online environments. Methodological design choices intended to improve equity and access for the target population may have an unintended consequence of improving access for fraudulent actors unless appropriate risk mitigation strategies are also employed. Lessons learned from this experience are likely to be valuable for novice health service researchers involved in online focus group discussions and interviews.

**Supplementary Information:**

The online version contains supplementary material available at 10.1186/s12913-024-11166-x.

## Background

Online recruitment and data collection have experienced significant growth in qualitative health services research, a trend driven in part by the COVID-19 pandemic [1, 2]. This shift to online methodologies has persisted due to the ease and convenience they provide for participant recruitment and data collection. Online approaches offer several benefits including flexible scheduling, elimination of travel expenses, reduced researcher and institutional influence, extended geographic reach, and engagement with ‘marginalised’ groups and participants often under-represented in research [3, 4]. Social media platforms such as Facebook and X (Twitter) have also broadened the scope of online recruitment by helping to engage potential participants with diverse demographics and across geographical regions [5, 6].

While the benefits of online recruitment and data collection in qualitative health services research are evident, this process also comes with notable challenges for both researchers and participants. For example, participants require technological proficiency, and researchers may experience challenges in obtaining consent, maintaining confidentiality, and interpreting limited body language cues [3, 4]. One of the less articulated issues of online recruitment and data collection is participant fraud or scam behaviour. The term **“imposter participants”** has been proposed to characterise individuals who, for the purpose of participating in research, provide false identities and experiences [7]. While participant fraud has been commonly observed in online surveys [7–10], its occurrence in online interviews is a relatively less common, although rapidly emerging phenomenon [11–14]. An increase in participant fraud has also been noted in online focus group discussions by qualitative market researchers, but has not yet been reported in health services research literature [15, 16]. The implications of fraudulent participation in qualitative research go beyond data invalidation and bias, as considerable ethical issues arise if an imposter participant takes part in a focus group discussion with genuine participants who share personal experiences.

We encountered one such case of participant fraud when dishonest participants were identified in online focus group discussions and interviews conducted as a part of a PhD health services research project. This research aimed to gather information from Australian families of children with neurodevelopmental disorders about important attributes of their follow-up care and experiences. The incident introduced methodological and ethical challenges into the PhD study. Although fraudulent participation caused disruption and delays in the study, we identified it at an early stage and implemented strategies to prevent this in further recruitment.

We aim to share our reflections on the imposter participant incident and the lessons learned specifically for early career researchers and online focus group discussions. Furthermore, we intend to provide recommendations for health services researchers engaging in online recruitment and data collection, particularly in fields related to paediatric neurodevelopmental care.

## Methods: our experience

### Participant recruitment

Participants were recruited online through an Australian neurodevelopmental organisation’s publicly available Facebook page where the study was advertised. The aim was to engage families of Australian children with neurodevelopmental disorders in either 90-minute focus group discussions or 30- to 45-minute individual interviews via an online communication platform, Zoom. A focus group/interview guide was developed for this study and is available as a supplementary file. A compensation of $50 AUD e-voucher was offered upon completion of the focus groups and interviews. The inclusion criteria were defined, and interested and eligible participants were encouraged to contact the research team via email for more information.

We received 254 expressions of interest within 24 hours of posting the advertisement. Due to practical limitations around sample size, purposive sampling was used to select participants across various locations within Australia. As per the locations provided by individuals in their expression of interest emails, we selected 25 participants for either a focus group discussion (four groups of five people each) or an individual interview (five participants) and provided them with a detailed participant information sheet and consent form. The selected individuals were sent a link to a brief Qualtrics survey to gather demographic information. For those not selected, a thank-you email was sent along with the option to express interest in future opportunities.

### Imposter participant suspicion and identification

On the day of the first focus group discussion, three out of the five participants joined the discussion. Although participants were encouraged to turn their cameras on, this was not required as a participation criterion. None of the attendees chose to turn on their cameras. During the discussion, one participant engaged verbally, while the other two requested to communicate via the chat function on Zoom. The team agreed on this preference considering their privacy and comfort. At the end of the discussion, we provided eGift cards (WISH gift voucher) to each participant. However, following the session, we received emails from all three participants, requesting Prezze eGift cards instead:

*Participant 1: “I appreciate your email. I was very busy to read my emails yesterday. The wish voucher you sent will be totally useless to me. I gave my reasons in the previous email I sent on Saturday or Sunday that I prefer a voucher that allows me to explore things I inted to buy. I feel I did the focus group for free without appropriate incentive. I suggested a preezze smart card which give me assess to multiple store of my choice or maybe an Amazon gift card but you didn’t take my opinion into consideration. I will appreciate while the gift card is still left un open to invalidate and cancel the card and send a better option. I will appreciate if you do that.”*

*Participant 2: “Thanks for your email. I didn’t even open the gift card until today. Like I said in the meeting that wish card is limited to me and I preferred the preezze that I could use to get other voucher. I feel very disappointed with the research team that after all the time I spent and the contribution I made I will be limited to the usage of the card. I only ask that the voucher be withdrawn and made invalidated and a better voucher be given. I feel that if a better voucher is not compensated I just made a free contribution without compensation.”*

*Participant 3: “Hi, I had requested that the card you sent be cancel and the money refunded back to you. This has been don as the balance on the card now remains zero. I will want an egift card from coles that I can easily use or Giftpay which I can select from a range of gift cards. Please look into this as soon as you can.”*

This type of specific request was new to the research team, so we considered the merit of honouring these requests because the participants contributed to the study. In this process, however, we discovered that the WISH gift cards we had offered were only valid within Australia, while Prezzee gift cards allowed international usage.

At this point, we also began to suspect that we had conducted a focus group discussion with not only imposter participants but also those posing as more than one person based on the similarity in the content of their emails. Given this finding, we analysed the initial participation request emails of these three focus group participants. All these emails revealed a strikingly similar textual pattern with minor variations (explained in detail in the Results section). Moreover, when using the IP addresses/geographical coordinates captured in the pre-participation Qualtrics survey as a cross-reference for the participants’ locations, we discovered that these locations did not align with the Australian states the participants claimed they were from. When using this technique to verify the location of all the other 22 selected participants, it became evident that none were genuine, and many responded from the same IP address/location. We also conducted two individual interviews on the same day as the focus group discussion. On comparing the recordings of these two participants’ voices, it was apparent that they were the same person. Both individuals also had the same geographical location, suggesting that they might be the same person assuming different identities. At this point, concerned about the ethical implications of imposter participants, we paused ongoing data collection and emailed the rest of the participants to cancel all scheduled interviews and focus group discussions. However, we still informed them that we were conducting a second round of screening due to the identification of fraudulent activity. If interested, they could fill out the survey again and provide their phone number to check for eligibility. Unfortunately, we received no response.

Given our suspicions, arising from a combination of the above-mentioned factors, that some of the initial contacts might be imposters, we initiated another round of screening to confirm this hypothesis across the broader sample. We contacted those individuals who expressed willingness to participate if not initially selected (*n* = 94). With a stricter eligibility criteria, we provided a Qualtrics survey link within an invitation email to proactively identify potential imposter participants at an earlier stage. We limited the survey to a single response and imposed Completely Automated Public Turing test to tell Computers and Humans Apart (CAPTCHA) settings to identify bots during survey completion. We also requested that participants provided their phone numbers to enable us to call them for further preliminary screening. Asking potential participants for phone numbers is an approach we have successfully used in the recruitment of families and carers previously and was also consistent with our ethical approval, which allowed us to contact participants in this way. Additionally, we specified that only WISH gift cards would be provided.

Despite these efforts, out of the 19 respondents (20% of all contacted) who expressed ongoing interest and completed the survey, none appeared to be genuine participants as their geographical details did not match those of the Australian states they claimed to be from. Two of them were identified as bot responses. Additionally, none of the participants supplied phone numbers for screening:

"*I would prefer an Amazon gift card. I prefer to participate through zoom call. I will be delighted to receive the zoom link from you.*"

"*Yes, I will participate, Was thinking how about we do every necessary enquiry here on email because the phone number here it’s a kind of family cell phone but I gain access to email thank you.*"

"*Yes, I will participate. Am currently not available in service, can we do a brief zoom call to go through the eligibility criteria?*"

Given these circumstances, we decided to terminate all further data collection from participants recruited via this process.

## Results: characteristics to identify fraudulent participation

Reflecting on our experience we have been able to highlight several key factors that pointed to the presence of imposter participants in our sample. It should be noted that the presence of one of these factors alone should not necessarily raise concern as genuine participants may exhibit one or more of these characteristics. Rather, a combination of several of these factors should alert suspicion.

### Factors that suggested fraudulent and/or duplicate identities

#### *Duration of focus group discussions and interviews*

The focus group discussion concluded within an hour, deviating from the scheduled 90-minute duration. The two individual interviews each lasted only 15 min, which was significantly shorter than the anticipated 30–45 min. Participants in the focus group discussion and the two interviews responded vaguely and lacked details, particularly about the hospitals they had visited or the doctors they had seen.

#### *Preference not to turn on the camera*

In both, the focus group discussion and interviews, none of the participants turned on their cameras. Additionally, two participants specifically requested to use the chat function on Zoom instead of joining the conversation verbally. This was an unusual occurrence for the experienced members of the research team. While we have previously encountered some families who may not wish to turn on cameras during interviews, it is infrequent. With the exception of studies where the target population may have verbal communication difficulties (e.g., among people with aphasia), none have expressed a preference to avoid verbal participation.

#### *Uncommon demographics*

An improbable observation was that nearly 80% of survey respondents self-identified as Aboriginal and Torres Strait Islanders. In contrast, 2021 data from the Australian Bureau of Statistics indicates that the Aboriginal and Torres Strait Islander people comprise only 3.8% of the total population [17]. Such a significant disparity raised concerns regarding data authenticity. Additionally, 80% of respondents indicated that their gender was male. While this could be attributed to chance, it is noteworthy that females typically outnumber males in general population demographics. Moreover, the experts on our team, who have previously conducted focus group discussions and interviews with a similar population, have usually received a majority of female (mother) participants. Previous research also confirms that greater proportions of mothers (compared to fathers) participate in research studies related to neurodevelopmental care for children [18–20].

#### *Unusual timings and large number of responses*

Receiving expressions of interest emails from individuals between midnight and 4 am AEST was concerning, given that we were recruiting Australian participants. Additionally, the amount of participant interest within 24 hours (*n* = 254) was unusual. It was noted that emails were sent promptly with a specific interval pattern. For example, ten emails were sent within one to two-minute interval, followed by a five to ten-minute break, and then another group of ten emails were sent within one to two-minute intervals.

#### *Confused participants*

Some individuals logged into Zoom with different names than those they registered with. They also tried to log in at times different from the scheduled time. This inconsistency could potentially be attributed to confusion arising from time zone differences. One interview participant struggled to identify the city within Queensland in which they were located. They only confirmed once we inquired if they were from Brisbane.

#### *Similarities in email addresses and writing patterns*

All these individuals used Gmail addresses, and a specific pattern was observed in their naming conventions. For instance, individuals with a first, middle, and last name structure (for example, ‘XYZ’) followed this pattern: the first three names followed by three numbers ‘xyz123@gmail.com’. Similarly, those with a first and last name format (for example, ‘AB’) adhered to a similar pattern, ‘ab12@gmail.com’. Another important observation involved the manner in which consent form signatures were provided by those who were selected for participation (initials with date; for example, PS − 13.10.2023).

Furthermore, the email patterns of potential participants exhibited similarities, with minor variations, as indicated in the text in Table 1.

**Table 1:** Email patterns of two potential participants with similarities

Participant 1	Participant 2
Introductory paragraph expressing interest	
*“I hope this email ****finds you well***. *I am writing to**** inquire about the possibility of participating****as a family member in the upcoming focus group session on neurodevelopmental follow-up care for children below 5 years of age with neurodevelopmental disorders and underlying medical conditions.”*	*“I hope this email**** finds you in good health***. *I am writing to**** express my interest in participating****as a family member in the upcoming focus group session for neurodevelopmental follow-up care for children below 5 years of age with neurodevelopmental disorders and underlying medical conditions.”*
Reasons to participate	
*“As a**** Father of a child who falls into this specific category***, *I have witnessed the importance of effective follow-up care in their overall development.**** My personal journey has provided me with valuable insights****into the challenges faced by families in accessing appropriate resources and support systems.**** I believe that participating in the focus group session would enable me to contribute****these insights and help shape the future of neurodevelopmental care for children with similar conditions.”*	*“As a**** parent of a child who falls within this demographic***, *I have**** firsthand experience of the challenges and unique needs****that arise when caring for children with neurodevelopmental disorders and underlying medical conditions.**** I strongly believe that my experiences, insights, and suggestions could contribute significantly****to the discussions held during the focus group session.”*
Previous experience	
*“I am**** deeply committed to advocating for the needs of children****with neurodevelopmental disorders and underlying medical conditions, and I actively engage with relevant communities and organizations. Through my involvement*, *** I have gained knowledge about various intervention strategies, therapies, and resources that can significantly benefit children in this demographic. Sharing this knowledge with other families and professionals****during the focus group session would provide a collaborative platform for improving the overall care and support available.* *I would be honored to be considered for participation and contribute to the important discussions that will take place.”*	*“I have**** actively engaged in seeking out the best possible care and support for my child***, *researching various interventions, therapies, and resources to ensure their optimal development. Through this journey*, *** I have gained valuable knowledge that I believe can be shared with other families and professionals****to enhance the quality of care provided to children in similar situations. Participating in the focus group session would not only give me an opportunity to share my experiences but also enable me to learn from other families and professionals. Collaboratively, we can work towards identifying gaps in the existing care system and explore innovative strategies to improve neurodevelopmental follow-up care for children in need.”*
Concluding paragraph	
*“**** If there are any additional requirements or information needed, please let me know. Thank you for considering my request***, *and I**** eagerly await the opportunity to be part of the focus group session**** on neurodevelopmental follow-up care for children below 5 years of age with neurodevelopmental disorders and underlying medical conditions.”*	*“I am available on the proposed date and time of the session and would be honored to be considered for participation.**** Should you require any further information or have any queries, please feel free to reach out to me. Thank you for considering my request***, *and I**** look forward to the opportunity to contribute to the discussion****on neurodevelopmental follow-up care for children below 5 years of age with neurodevelopmental disorders and underlying medical conditions.”*

*Bold = highlighted to indicate the similarity in text

#### *Inappropriate and duplicate locations*

**Fig. 1:**
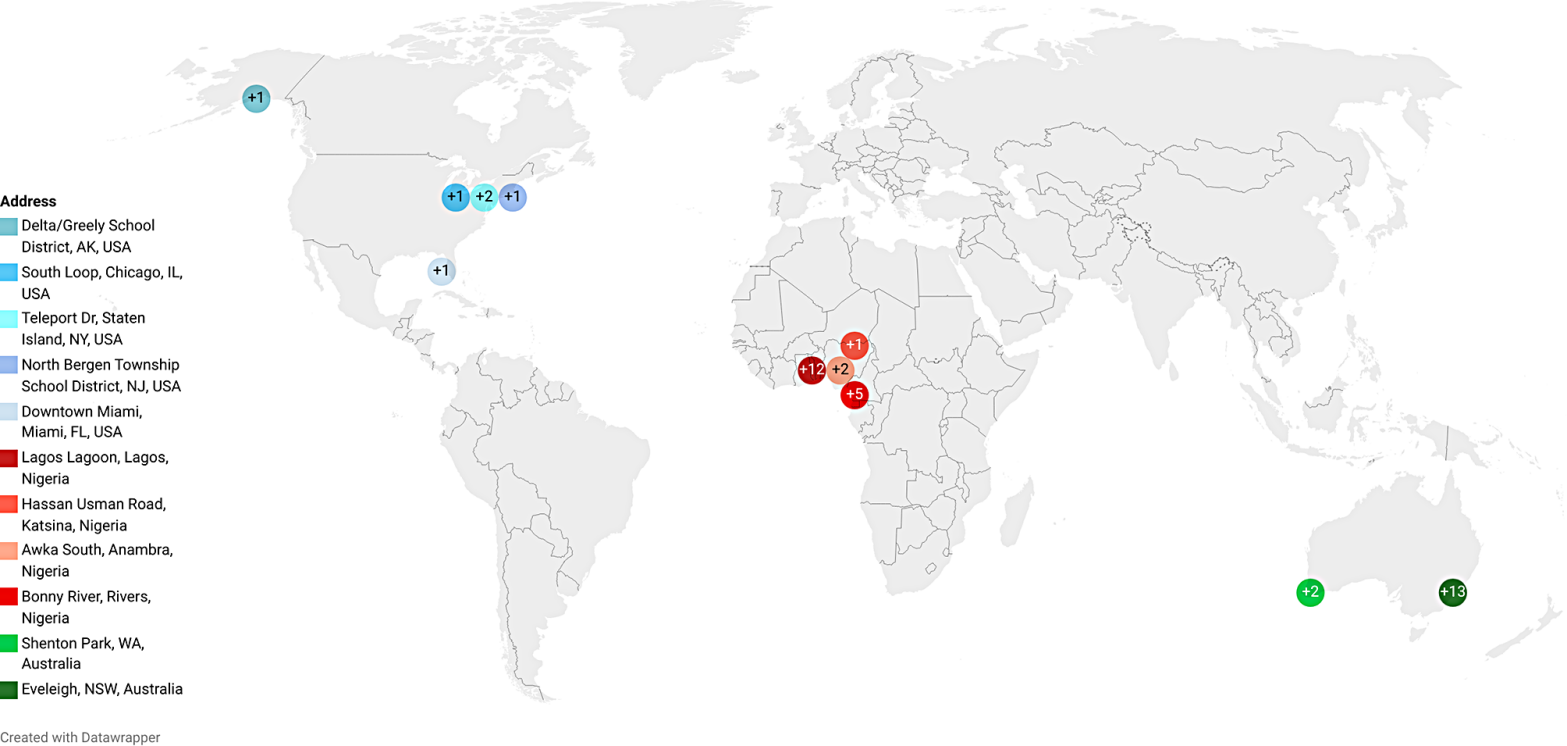
Location of the individuals who completed the survey. Image was created with the DataWrapper online tool

As described, inconsistencies in a participant’s reported location and that of their IP address were important red flags for highlighting imposters. Figure 1 provides a visual representation of the survey completion locations in our sample. IP addresses indicated that respondents were located in Nigeria (*n* = 20), Australia (*n* = 15), and the United States (*n* = 6), although the use of location spoofing was suspected. Similarly, individual participants may have attempted to use multiple fraudulent identities.

### Observed differences between imposters and genuine participants

After informing the neurodevelopmental organisation through which we recruited about the incident, they advertised our study again in their monthly newsletter and closed Facebook page with nearly 3000 screened members only. We maintained a stringent eligibility confirmation process, which included turning on cameras at the start of the interview or providing legitimate Australian phone numbers. As a result, we conducted two individual interviews (both with female participants). After speaking to the genuine participants, significant differences were observed in their responses when compared to imposter participants. Their interviews extended for a complete 45-minute duration or longer, and they adhered to the requirement of turning their cameras on. Their answers exhibited a high level of specificity and detail. For example, they referred to their children by their names and specified care provider details. Genuine participants never inquired about the gift cards, whereas imposters asked multiple times before and during their interviews.

## Discussion

Our experience and the suggested indicators of fraudulent participation align with findings from other recent studies using qualitative methods. These indicators include a high volume of emails sent in a short time and unusual timing of emails [13, 14], similarities in content and email address format [12], preference for not turning on the camera [11], vague and inconsistent participant responses, as well as signs of confusion [10, 21], and potential financial interests [7].

When researchers rely on participant self-reports for determining eligibility, the quality and validity of the collected data depends upon the honesty of the participants [7]. However, employing self-reports in our study as the only means of verification posed limitations, a situation also encountered by another PhD researcher [11]. While the intention was to prioritize participant privacy, it created a possibility for imposters to participate. Recently, other commentaries and reports have also highlighted similar experiences [14, 21–24]. For example, an autism study reported that some participants identified themselves as autistic individuals or parents of autistic children, possibly for monetary gain [22]. Researchers observed comparable issues including short duration of interviews, unlimited participant availability, apparent unclear responses, frequent questions regarding receiving compensation, and inconsistencies in their reported locations.

There are several options that may help to combat the risk of fraudulent participants taking part in qualitative health services research. Others have suggested that participants should be required to provide legitimate mobile numbers for a pre-screening phone interview, as long as this aligns with the project’s ethical approval [12, 22]. This approach was successfully adopted in our second round of screening. The possibility of requesting a government identity should also be explored [22]. Some researchers suggest not disclosing the compensation amount [14, 23], while others suggest not providing compensation at all [22]. However, this strategy may have disadvantages including discouraging genuine participants and not valuing their time and effort. Additionally, advertising in closed social media groups may be feasible, a strategy successfully employed by our study and another [6]. Even though ‘open’ recruitment approaches (e.g., Facebook public page posts, large online communities, or other ‘open’ research invites) may intend to improve access to participants, their broad reach and public availability increases the risk of imposter participants being recruited. If applicable, turning on cameras should be mandatory, even if it is for a few seconds, to avoid including one person with multiple personas [12, 25]. Other suggestions for identifying ‘red flags’ include screening email content if similarities are observed, being alert if a large number of emails are received in a short span of time, being attentive to excessively quick and vague responses, and repeating questions in different ways related to the inclusion criteria [11, 12, 21]. A commentary suggested that genuine participants usually write about why they would like to participate [23]. However, this is contradictory in our study, as imposter participants provided lengthy messages while the genuine ones only provided one sentence expressing their interest. Although we did not reach the data analysis stage due to the timely identification of imposters, instances of fraud may still occur even with strict criteria, and researchers should remain cautious even when analysing data. This may include observing responses for inconsistencies and cross-checking answers with self-reported information or survey data [11, 12].

### Lessons learnt and recommendations

With the recent increase in such fraudulent activities, research integrity is at stake, demanding immediate action. While we agree with the strategies already suggested by others to combat this issue (see above), we feel that it is imperative to share additional recommendations based on our unique experience, particularly to avoid the inclusion of single imposter participants posing as multiple people.

During recruitment:

- An initial step to confirm participants’ identity and location can be incorporated. Tools such as Qualtrics can be used specifically for location verification.
- The ethnicity, cultural and gender demographics of a focus group discussion or interview sample can be compared to the region’s general statistics.
- Apart from email patterns, small details such as signature styles on consent forms can be checked.
- It can be indicated in the consent form that compensation will not be provided if fraud participation is detected. However, this should be declared in the ethics protocol.
- The types of eGifts to be used can be clearly communicated and emphasis can be placed on the limited options available. Researchers should be suspicious if participants request a different gift card.
- Minimal information that may be necessary for verification can be obtained and disclosed in the consent form and ethics. For example, in our research study, we could have asked participants to identify the hospital or service they accessed.
- Institutions and ethics committees can provide learning modules to early career researchers, enabling them to be alert and equipped to handle such situations. Additionally, ethics protocols can include a section on how researchers should address such issues if they arise in their study.

During the focus group discussion/interview:

- Typing comments instead of supplying them verbally during sessions can be restricted to enhance the authenticity of responses. There is no assurance that the participant typing is the intended individual. It is also possible that they may be using Artificial Intelligence or other technology to generate answers [26]. Moreover, in qualitative studies, researchers maybe interested in observing spontaneity that accurately represents a participant’s thoughts or feelings, which cannot be achieved via text responses.
- Researchers should become alert if individuals are logging in at different times or with different names.
- Participants can be prevented from joining from other countries during Zoom conferencing. This can be done via ‘settings’ option in Zoom.

Even after implementing preventative measures, it is possible that imposters may pass the recruitment stage and participate in interviews. While we have outlined recommendations that may prevent imposters from joining focus groups or interviews, it may be challenging to tackle the issue if they have only been identified in the middle of an ongoing discussion. In this case, others have suggested the interviewer ask a new or unexpected question that was not originally in the interview guide [22], or asking probing questions if responses seem unclear [11]. Given these circumstances, it is crucial for the researchers to safeguard both themselves and genuine participants. In such situations, researchers could politely ask suspected imposters to leave the discussion using phrases like, “Thank you for your participation, that was all the information we required at this stage, we will follow up with you later.” Alternatively, researchers could end the entire focus group, asking all participants to politely leave. Suspected imposters may be contacted for further clarification and confirmation of identity. Researchers should also follow up with genuine participants to brief them on the situation and assure them that their data will not be used if they are uncomfortable or if they wish to reschedule the focus group. Additional support, including free counselling available at researchers’ institution, may also be offered. However, all these approaches should be discussed with the institutional ethics board before implementation.

### Limitations and ethical considerations

The aforementioned recommendations are informed by both experiences in the present study, as well as prior literature, and are likely to be beneficial for the detection of imposter attempts similar to those experienced in the present study. However, these recommendations should be considered a useful starting point, rather than comprehensive guidance. This is particularly the case for imposter attempts from bad actors who may have access to more sophisticated technologies for spoofing identities and locations. There are likely to be many benefits from the democratisation of generative artificial intelligence, including large language models capable of rapidly producing compelling written prose, or video and image filtering or generation; however, one of the potential undesirable consequences may include greater difficulty in detecting imposter participants with easily implementable strategies.

There are also a range of ethical considerations regarding the publication of information related to imposter participants. Of primary concern, we felt an obligation to report these findings and recommendations to raise awareness of this issue in the context of qualitative methods to promote and support research integrity in the health services research community. We agree with Santinele Martino et al. [27] that there appears to be a growing and well-organised industry of fraudulent research participants impacting research across multiple counties which must be highlighted. However, we are also mindful that the open publication of these experiences and recommendations may contribute to the evolution of fraudulent strategies by bad actors seeking to deceive researchers through imposter participation. On balance, we considered the potential benefit to the community from reporting these experiences openly to outweigh the potential risks associated with disclosing these recommendations.

## Conclusion

Although online qualitative research and data collection have advantages for health service researchers and participants, the rapid adoption of this technology has increased the risk of encountering imposter participants. Our experience highlights the importance of thorough recruitment practices and maintaining alertness during online data collection to maintain research integrity. Despite these challenges, we have derived valuable lessons from this experience. We emphasize the importance of perseverance during such setbacks and acknowledge that navigating such complex situations is an inherent part of research and may not always be easily predicted or prevented.

### Electronic supplementary material

Below is the link to the electronic supplementary material.

Supplementary Material 1

## Data Availability

The datasets generated and/or analysed during the current study are not publicly available due to privacy considerations as they may contain potentially identifiable information about participants but are available from the corresponding author on reasonable request.
