# Supplementary material for: Navigating the challenges of imposter participants in online qualitative research: lessons learned from a paediatric health services study
Source: BMC Health Serv Res. 2024 Jun 12;24:724. doi: 10.1186/s12913-024-11166-x (PMC11170877; doi:10.1186/s12913-024-11166-x)
Supplement: Supplementary file 1 — Supplementary Material 1 [file 12913_2024_11166_MOESM1_ESM.docx]

**Preferences for a neurodevelopmental follow-up care for children**

**Interview / focus group discussion guide**

**Objective:**

The objective of the focus group/interview is to identify and explore potential attributes (characteristics) neurodevelopmental follow-up care for children, and their associated levels with key stakeholders.

**Participants:**

Family members (including parents, siblings, and/or caregivers) of children below 5 years of age diagnosed with neurodevelopmental disorders with an underlying medical history such as congenital heart disease, surgeries, and prematurity.

Potential participants will be identified through social media handles of organizations involved with neurodevelopmental follow-up care for children. Interested individuals will be asked to directly contact the research team through the link provided in the invitation. When individuals write to the research team expressing their interest in participation, they will be contacted by a research team member through a return email/telephone call. During this communication process, the interested individuals will be screened for eligibility criteria mentioned in the protocol, and other required information such as current follow-up care, previous hospital admissions, other comorbidities, geographic location and socio-demographics will be obtained to ensure the maximum variation sampling to allow a variety of viewpoints. The interested individuals will be sent the consent form to be signed and returned to the research team.

Each focus group/interview should be no longer than 90 minutes in length, with a maximum of 10 participants.

**How to use this guide:**

The questions are based on a review of relevant literature and experts opinion to ensure all aspects of evaluation are considered in a comprehensive manner.

1. All key questions should be covered in the focus group. However, it is not necessary for interviewers to ask questions in the proposed order or repeat a question if it has been answered in an earlier section.
2. The conversation should explore issues as they are raised, with guidance and prompting as required.
3. Facilitators will ensure discussion progresses in a timely, yet informative manner.

**Focus group/interview script:**

*This section is included as an example of introductory text for the focus group/interview. Please note this can be adjusted and amended to suit the interviewer, context, or format of delivery.*

Hi, welcome to this focus group discussion/interview exploring attributes for neurodevelopmental follow-up care for children. Thanks for giving us your time today. My name is Pakhi. I am a researcher with Australian Centre for Health Services Innovation, Queensland University of Technology. The purpose of our discussion is to understand and explore the different attributes and preferences of effective, accessible, and acceptable neurodevelopmental follow-up care for children. Our discussion today will last about 90 minutes.

First, I would like to know how you all are feeling right now. Does any of you feel uncomfortable or ill right now? (Make sure everyone responds). Thank you.

This discussion that we wish to have with you today is part of a bigger project that is aimed at understanding preferences for a neurodevelopmental follow-up care for children. The use of the term “neurodevelopmental follow-up care” refers to the care provided to children and their families over time after diagnosing the neurodevelopmental disorder or delay in order to monitor the medical and developmental outcomes and therefore provide suitable support. As key stakeholders who have received or are receiving follow-up care, it is important that your views are taken into consideration when understanding the key attributes of an ideal follow-up care.

This research may not directly be beneficial for you, but we hope the results of this study will be influential in changing the neurodevelopmental follow-up care for children positively to provide them with better health care. We would like to find out your opinion, specifically, we will discuss the following issues with you:

- attributes of current and ideal follow-up care for children with neurodevelopmental needs.
- acceptable or expected levels/parameters of the attributes discussed today.
- facilitators and barriers of neurodevelopmental follow-up care for children.

We will record the conversation with you if that is OK, and we will only share your conversation with other members of our research team by making sure other people cannot identify your responses as an individual. It is important that everyone here feels that this is a safe space to share, so it is important to allow all participants the chance to speak, and to respect each other’s right to express different opinions, even when you do not agree with them. Also, we would like to request from you to keep everything we discuss here in confidence and please do not mention names of practitioners or institutions when you express your thoughts.

We have obtained ethical clearance for this study from Human Research ethics Committee, Queensland University of Technology.

We really appreciate your time and willingness to speak with us. Does anyone have any questions before we get started?

**Interview key questions:**

1. What are your experiences with neurodevelopmental care services?

Prompts:

- What are some of the challenges/barriers?
- What are some of the positive experiences?
- What kind of care providers have you consulted? For example, GPs, paediatricians, allied health services, etc.

1. How do you feel about the current health system and what could be improved in the current system?

Prompts:

- Do you prefer services to be closer to home or is it fine to travel?
- Is care easily accessible?
- How much do you usually wait for an appointment?
- Do you receive any information regarding the diagnosis of your child? For example, epidemiology (cause of problems and what to expect as child gets older), developmental milestones, diagnoses, treatment options, and service information. And what are some of the ways you would like to receive this information? For example, group activities or peer support groups, one-on-one with a professional, online learning material, etc.
- Do you prefer or already receive mental health support?
- What are your thoughts on telehealth/online follow-up system?

1. Imagine you are running the health system, you have unlimited budget/money, how would you deliver care/manage the system?

OR

What is an ideal neurodevelopmental follow-up care according to you?

- How would you like the cost of screening/assessments/interventions to be?
- Will the waiting time be reduced?
- Will you appoint more specialized care providers?

1. Is there anything else you wanted to say about neurodevelopmental follow-up care for children and your experiences with it?

We have now come to the of our discussion. I would like to summarise the opinions expressed here for each question now. Please take a 10-minute break until I prepare the summary to be shared with you.

**(10 minutes break)**

Thank you for your patience. The summary of the ideas expressed here are as follows. (Presents the summary). I’d like to know if anyone disagree with this summary of opinions.

Thank you very much for your time today and we really appreciate your contribution to this research. We hope your efforts will contribute to improve neurodevelopmental follow-up care for children. Thank you and have a good day!

**End of guide**
